# Orthodontists’ preferences regarding the difference of bracket bonding height between the maxillary incisors

**DOI:** 10.1590/2177-6709.26.6.e212031.oar

**Published:** 2021-12-15

**Authors:** Mauro Henrique Andrade NASCIMENTO, Karla Magnólia Napoli BRANDÃO, Carolina Carmo de MENEZES, André Wilson MACHADO, Milton SANTAMARIA-JR

**Affiliations:** 1 Programa de Pós-graduação em Ortodontia do Centro Universitário da Fundação Hermínio Ometto-FHO (Araras/SP, Brazil); 2 Universidade Federal da Bahia, Faculdade de Odontologia, Departamento de Ortodontia (Salvador/BA, Brazil)

**Keywords:** Dental esthetics, Smile, Orthodontics

## Abstract

**Introduction:**

The vertical position of orthodontic brackets in maxillary incisors may influence the incisal step between the anterior teeth and thereby interfere with the smile esthetics. Even so, esthetic standards have been modified over time and consistently required technical adjustments.

**Objective:**

This study analyzed orthodontists’ preferences regarding the difference of bracket bonding height between the maxillary central incisors (MCI) and maxillary lateral incisors (MLI), and further determined whether the orthodontist sex, age and time of specialization have association to their choices.

**Methods:**

This study collected data through an electronic form. Study participants analyzed a clinical case in which they indicated their preference for bracket bonding height. The placement height options ranged from 3.0 mm to 5.5 mm from the incisal edge, with 0.5-mm intervals, or at the clinical crown center (CCC). The difference in the bonding height between the MCI and MLI was analyzed, considering the formation of incisal steps between these teeth.

**Results:**

Participants indicated that the difference in bracket bonding height between the MCI and MLI should be as follows: 0 mm (3.9%); 0.5 mm (78.3%); 1 mm (7.6%); 1.5 mm (0.2%); and CCC (9.9%). There was no statistically significant correlation between the choice for bracket bonding height and sex, age and time since specialization.

**Conclusion:**

Most participating orthodontists choose the 0.5-mm difference in bracket placement height between the MCI and MLI. The variables sex, age and time since specialization did not influence this choice.

## RESUMO

**Introdução:**

O posicionamento vertical dos braquetes ortodônticos dos incisivos superiores influencia diretamente no degrau formado entre esses dentes, podendo interferir na percepção estética do sorriso. Além disso, os padrões estéticos se alteram ao longo do tempo, demandando ajustes nas técnicas para acompanhá-los.

**Objetivo:**

O presente estudo analisou as preferências dos ortodontistas em relação à diferença na altura de colagem dos braquetes entre os incisivos centrais superiores (ICS) e os incisivos laterais superiores (ILS) e determinou, ainda, se o sexo do ortodontista, idade e tempo de especialização têm associação com suas escolhas.

**Métodos:**

A pesquisa foi realizada por meio de formulário eletrônico incluindo as variáveis sexo, idade e tempo de formação profissional. Em seguida, os participantes analisaram um caso clínico, para o qual indicaram as alturas de colagem dos braquetes que aplicariam, com as opções de 3,0 mm a 5,5 mm da borda incisal, em intervalos de 0,5 mm; ou no centro da coroa clínica (CCC). Os dados foram analisados utilizando-se as diferenças entre as alturas de colagem dos ICS e ILS, refletindo na formação de degraus verticais entre esses dentes.

**Resultados:**

Encontrou-se que os profissionais utilizariam a seguinte diferença de altura na colagem dos braquetes de ICS e ILS: 0 mm (3,9%); 0,5 mm (78,3%); 1 mm (7,6%); 1,5 mm (0,2%); e CCC (9,9%). As correlações entre a preferência das alturas de colagem e o sexo, idade e tempo de formado não foram estatisticamente significativas.

**Conclusão:**

Na amostra pesquisada, existiu uma preferência pela diferença de altura na colagem entre ICS e ILS de 0,5 mm, sendo que os fatores sexo, idade e tempo de formação não tiveram influência na escolha.

## INTRODUCTION

An appropriate positioning of orthodontic accessories is required for alignment and leveling of dental arches, which is a basic precept of orthodontic intervention. Hence, minor variations in the positioning of orthodontic brackets and other accessories may jeopardize the efficacy of the orthodontic treatment.^1^ ^-^ ^3^ The bonding height of anterior brackets has a high impact not only on establishing overbite and mandibular function, but also on the vertical position of the incisors, which ultimately reflects on aspects such as youthfulness, sensuality and smile esthetics.^4^ ^-^ ^9^ The demand for esthetic treatments has increased greatly the last decades.^7^ ^,^ ^10^ Orthodontists should be aware that the correct placement of brackets may vary in each patient, which should be accounted for in the orthodontic and esthetic planning.^11^ ^-^ ^14^

Several orthodontic prescriptions have been suggested over the years, with some variation of protocols and techniques regarding bracket bonding heights. Most authors propose that orthodontists should keep a difference between 0.0 mm and 0.5 mm in the bonding height of orthodontic brackets between the maxillary central incisors (MCI) and lateral incisors (MLI), which may affect the step between these teeth and the smile arc.^11^ ^,^ ^13^

Studies analyzing the esthetic perception of incisal steps between the MCI and MLI and their influence on the smile arc have shown that orthodontists frequently fail to follow the recommended bracket bonding heights prescribed in the literature.^6^ ^-^ ^8^ ^,^ ^13^ However, orthodontists’ preferences regarding the difference of bracket bonding height between the MCI and MLI remain unknown.

Despite the variations in tooth anatomy, most orthodontic prescriptions are based on population averages, have disregarded the smile arc, are relatively old, and have not been adapted to current esthetic requirements.^13^ Thus, the present study aimed to analyze the difference in bracket bonding heights between the MCI and MLI, which is responsible for the central-to-lateral incisal step at the end of the alignment and leveling phase. Furthermore, the variables sex, age and time since specialization were checked for an association with orthodontists’ preferences, and the results obtained herein were compared against the esthetic standards reported in the literature.

## MATERIAL AND METHODS

This study was previously approved by the Research Ethics Committee of *Faculdade de Odontologia da Universidade Federal da Bahia* (Salvador/BA, Brazil), under protocol CAEE 98475118.4.0000.5024. The sample size was calculated, considering a finite population (n = 16.000), with 95% confidence interval, normal quantile of 1.96, maximum allowable error of 5% (*p* = 0.05), variance of 0.25 and sample power of 80%. So the sample size (n) was established in 432 participants. 

An electronic form (Google forms) was sent by email and via a text message app, to a database of approximately sixteen thousand orthodontists in Brazil (n = 16.000), including orthodontists enrolled at the Federal Council of Dentistry, and graduate students in Orthodontics. A total of 467 forms were returned, which underwent a data consistency analysis to exclude duplicate information and possible coding errors in the data electronic form re-sent to the researcher, totaling a final sample of 434, a final response rate of 2.71%.

**Figure 1:**
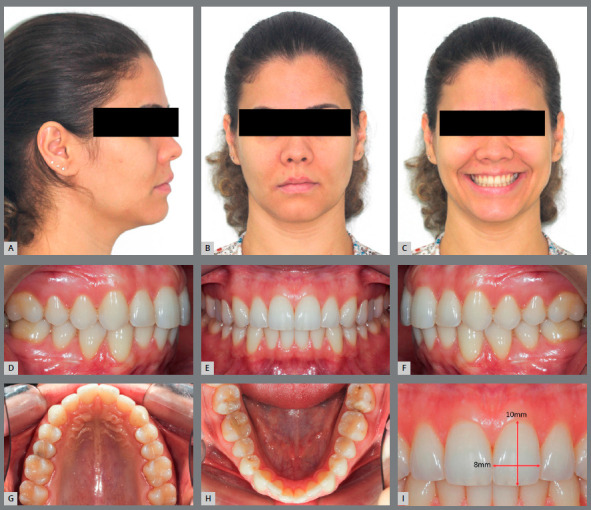
A-H) Extraoral and intraoral photographs of the case. I) Height / width ratio of the maxillary central incisors.

The electronic form contained questions related to sex, age and time since specialization training in orthodontics. The variables sex, age and time since specialization were checked for any correlation with the orthodontists’ preferences regarding bracket bonding height of the maxillary incisors teeth. The study participants were asked to evaluate clinical parameters - facial photographs (frontal, smile and right-side profile), intraoral photographs (frontal, right-side and left-side lateral, and occlusal) and a photograph indicating the length and width of the MCI clinical crown (Fig 1).

The clinical parameters presented a female patient with Angle Class I malocclusion, absence of crossbite, 30% overbite, absence of significant dental asymmetries, and slight mandibular crowding. The patient had 3-mm passive exposure of the MCI, 0-mm smile gingival exposure when smiling, and a nice smile arc, as normal clinical parameters.^10^ ^,^ ^15^ ^-^ ^19^ In the intraoral photographs, the size of the incisors was adjusted for an 80% width/height ratio. This adjustment aimed to avoid bias while determining the bracket placement height, with a potential need for gingival recontour or increment of incisal edges. In addition, one of the sides was mirrored to avoid asymmetries.^20^ All adjustments were carried out using Adobe Photoshop (version CS5; Adobe Systems, San Jose, California, USA).

This study was based on previous researches, in which it was found that the 2-mm central-to-lateral incisal step was considered aesthetic^6^ ^,^ ^7^ ^,^ ^8^. The participants were asked to indicate their choice of bracket bonding height for the maxillary incisors and canines, among one of the following possibilities: 3.0 mm to 5.5 mm from the incisal edge, with 0.5-m intervals, or at the clinical crown center (CCC). As one of the arch sides was mirrored, the participants were asked to choose only one placement height option for the MCI, MLI, and canines, regardless of the arch side. Irrespective of the height option chosen by each participant, the analysis considered the difference in bonding height between the MCI and MLI, which resulted in one of the following possibilities: 0.0 mm, 0.5 mm, 1.0 mm and 1.5 mm, or CCC. The placement height preferences for the maxillary incisors and canines were analyzed, but only the MCI and MLI heights were considered in this study.

### STATISTICAL METHODOLOGY

After sample size was calculated, based on a pilot study, and the data reliability was analyzed, the demographic aspects (sex, age, time of specialization training) and the bracket bonding height difference between the MCI and MLI were analyzed as independent variables, expressed in absolute and percent values.

In inferential statistical analysis, bracket bonding height difference was considered the study outcome variable; and demographic aspects (sex, age and time of specialization training) were the independent variables. After analysis of data consistency, for the hypothesis test, Chi-square was used to check for an association between the demographic aspects and the bracket bonding height preferences. Data were analyzed using R software (version 3.5.1,R Foundation for Statistical Computing, Vienna, Austria), considering a significance level of 5%.

## RESULTS

The sample characteristics are presented in Table 1. The sample presented 54.4% of women and 45.6% of men, with the age varying by 60.6% between 30 to 50 years old. The time since specialization training was between 5 to 15 years in 39.9% and 15 to 25 years in 21.7% of orthodontists. The less experienced participants - graduate student and with less than 5 years of graduation - accounted for 28.8% of the sample. 

**Table 1:** Descriptive analysis of the demographic variables.

Variable	n	%
Sex		
Female	236	54.4
Male	198	45.6
Age range (in years)		
20 to 30	84	19.4
30 to 40	121	27.9
40 to 50	142	32.7
50 to 60	62	14.3
Older than 60	25	5.8
Time since specialization training (in years)		
Ongoing (graduate student)	42	9.7
Less than 5	83	19.1
Between 5 and 15	173	39.9
Between 15 and 25	94	21.7
More than 25	42	9.7

Table 2 shows the absolute and percent values of the bonding height differences between the MCI and MLI brackets. There was a predilection for the 0.5-mm difference by 78.3% of the study participants. In addition, there was the choice for 1-mm bonding height differences between the MCI and MLI brackets (7.6%) and 9.9% of orthodontists prefer the bonding of the maxillary incisors on CCC.

**Table 2:** Absolute and percent values of the difference in bracket bonding heights between the maxillary central and lateral incisors.

Bracket bonding height difference between the MCI and MLI	n	%
0.0 mm	17	3.9
0.5 mm	340	78.3
1.0 mm	33	7.6
1.5 mm	1	0.2
CCC	43	9.9

Note: MCI = Maxillary Central Incisors; MLI = Maxillary Lateral Incisors; CCC = Clinical Crown Center.

As shown in Table 3, the variables sex, age and time since specialization were not significantly associated with orthodontists’ preferences regarding the difference of bracket bonding height between the MCI and MLI (*p*> 0.05). In other words, regardless of sex, age and specialization training time, professionals determined a 0.5-mm step between MCI and MLI, and the biggest difference of 1.5mm in bonding was the least preferred.

**Table 3:** Association between the study variables and the difference in bracket bonding heights between the maxillary central and lateral incisors.

Variable	Bracket bonding height difference between the MCI and MLI					P-value ^1^
	0.0 mm n (%)	0.5 mm n (%)	1.0 mm n (%)	1.5 mm n (%)	CCC n (%)	
Sex						
Female	8 (3.4)	188 (79.7)	20 (8.5)	1 (0.4)	19 (8.1)	0.457
Male	9 (4.5)	152 (76.8)	13 (6.6)	0 (0.0)	24 (12.1)	
Age range (in years)						
20 to 30	3 (3.6)	71 (84.5)	6 (7.1)	0 (0.0)	4 (4.8)	0.431
30 to 40	3 (2.5)	92 (76)	12 (9.9)	1 (0.8)	13 (10.7)	
40 to 50	4 (2.8)	114 (80.3)	8 (5.6)	0 (0.0)	16 (11.3)	
50 to 60	4 (6.5)	44 (71)	5 (8.1)	0 (0.0)	9 (14.5)	
Older than 60	3 (12.0)	19 (76.0)	2 (8.0)	0 (0.0)	1 (4.0)	
Time since specialization training (in years)						
Ongoing	3 (7.1)	32 (76.2)	2 (4.8)	0 (0.0)	5 (11.9)	0.407
Less than 5	1 (1.2)	70 (84.3)	9 (10.8)	0 (0.0)	3 (3.6)	
5 to 15	4 (2.3)	134 (77.5)	14 (8.1)	1 (0.6)	20 (11.6)	
15 to 25	6 (6.4)	72 (76.6)	4 (4.3)	0 (0.0)	12 (12.8)	
More than 25	3 (7.1)	32 (76.2)	4 (9.5)	0 (0.0)	3 (7.1)	

^1^ Chi-square test. MCI, Maxillary Central Incisors; MLI, Maxillary Lateral Incisors. CCC = Clinical Crown Center.

## DISCUSSION

The central-to-lateral incisal step can be formed in the fixed orthodontic therapy by placing brackets at different heights or by making intrusion and extrusion bends. While studies on the esthetic preferences of orthodontists have been recently published,^6^ ^-^ ^8^ ^,^ ^13^ there are no population-based studies addressing orthodontists’ preferences in the difference of bracket bonding height between the maxillary incisors, as analyzed herein.

The bracket bonding height in maxillary anterior teeth can highlight the central incisors incisal edges by establishing a greater or smaller step with the lateral incisors, as well as it can affect the smile arc design, a primary feature in dentofacial esthetics. In the present study, most orthodontists chose the 0.5-mm bonding height difference between the MCI and MLI, which directly affects the step formed between the incisal edges of these teeth that have the most significant impact on the smile esthetics and convexity of the smile arc.^6^ ^,^ ^8^ ^,^ ^13^ ^,^ ^21^ These findings are consistent with most prescriptions described in the literature^11^, which seems reasonable, since specialization students in orthodontics are expected to be taught what has been consolidated in the literature.

Nevertheless, recent studies on smile esthetics have shown that both orthodontists and laypeople prefer central-to-lateral incisal step of 1.0 to 2.0 mm.^6^ ^,^ ^8^ ^,^ ^21^ Changes in bracket bonding heights have a direct influence on the gingival design, but this fact has less esthetic importance compared to the step formed between the incisal edges of the MCI and MLI.^6^ The present study indicates that 78.3% of the participants selected the 0.5-mm difference in bracket bonding height between the MCI and MLI, and that 3.9% of them would bond the brackets at the same height in both teeth. This totals 82.2% of the sample that would adopt measures that would create steps different to the 1.0 to 2.0 mm values recommended by recent studies on smile esthetics.^6^ ^,^ ^8^ ^,^ ^21^

The orthodontists who place the orthodontic brackets at the CCC level commonly do so in all teeth. While they are not necessarily looking for a more esthetic relationship between the MCI and MLI, this could create a step of approximately 0.8 mm and 0.9 mm for males and females, respectively, considering the average size of the maxillary anterior teeth.^22^ Therefore, this specific parameter is closer to the esthetic values described in the literature.^6^ ^,^ ^8^ ^,^ ^21^ It is worth noting this would be beneficial only for teeth with an average anatomical proportion.

The clinical parameters example used in this study was of a female patient. Some authors point out that females should have a greater step in the maxillary incisors and, consequently, a more pronounced smile arc,^6^ ^,^ ^8^ ^,^ ^23^ which could lead to a bonding height preference with a step greater than what was found. Considering that orthodontists tend to prefer the 1.0-to-2.0-mm step between the MCI and MLI,^6^ ^,^ ^8^ ^,^ ^21^ there seems to be inconsistency between such esthetic preference and their bracket bonding height choice.

The results obtained herein are compatible with those of the main prescriptions for bracket positioning.^11^ However, this may mislead most orthodontists to place orthodontic accessories in an inconsistent position with that for incisors they would like to obtain. Therefore, the orthodontists’ esthetic preferences differing from their option for the bracket bonding heights, led us to hypothesize that three situations are likely to occur, namely: (i) completion of orthodontic treatment with incisal steps smaller than desired, which may be a result of the lack of esthetic perception regarding the step between the MCI and MLI; (ii) rebonding of maxillary anterior brackets; or (iii) intrusion or extrusion bending between the MCI and MLI to obtain the desired esthetic outcome.

Extrusion or intrusion bending of incisors, or bracket rebonding for this purpose, require contention of orthodontic movements for better stability, particularly prior to removal of the fixed orthodontic appliance. Thus, the execution of rebonding or bending procedures near the removal of the orthodontic appliance may cause relapses and major esthetic losses.^24^ ^,^ ^25^ Anterior and laterality guides should also be checked while changing the steps between the incisors.^6^ ^,^ ^26^

The present study showed the variables sex, age and time since specialization training were not significantly associated with the orthodontists’ preferences in the difference of bracket bonding height between the maxillary incisors. So, this study rejects the hypothesis that younger or recently graduated professionals would be more likely to adopt a bracket placement height consistent with current esthetic standards.

In this research, the number of forms submission to the orthodontists was large, however there was a small return compared to the total, although safe statistical calculations were possible. This is a difficulty common to surveys that carry out data collections through questionnaires. So, this study did not aim to indicate the best bracket placement height for maxillary anterior teeth, but only to compare orthodontists’ preferences with what has been recommended in the literature for esthetic design for central-to-lateral incisal step, in this studied population. Variations in the sample, dental anatomy, or statistical modeling are likely to occur, which makes it inappropriate to generalize the measurements obtained herein. Further studies are needed to better understand the relationship between orthodontists’ preferences regarding bracket bonding heights and the current esthetic standards of maxillary anterior teeth vertical positioning.

## CONCLUSION

Based on the findings obtained and the limitations of the study, it may be concluded that:

» Most orthodontists chose the 0.5-mm difference in the bracket bonding height between the MCI and MLI.
» Only 7.8% of them designated bonding height differences between the MCI and MLI of 1.0 mm or 1.5 mm.
» Orthodontist’s choices regarding bracket bonding heights were not affected by sex, age and time since specialization training, in this population.
